# The Inhibition of N-Glycosylation of Glycoprotein 130 Molecule Abolishes STAT3 Activation by IL-6 Family Cytokines in Cultured Cardiac Myocytes

**DOI:** 10.1371/journal.pone.0111097

**Published:** 2014-10-23

**Authors:** Reo Matsuo, Hirofumi Morihara, Tomomi Mohri, Shiho Murasawa, Kana Takewaki, Hiroyuki Nakayama, Makiko Maeda, Yasushi Fujio

**Affiliations:** Graduate School of Pharmaceutical Sciences, Osaka University, Suita City, Osaka, Japan; National Cancer Institute, United States of America

## Abstract

Interleukin-6 (IL-6) family cytokines play important roles in cardioprotection against pathological stresses. IL-6 cytokines bind to their specific receptors and activate glycoprotein 130 (gp130), a common receptor, followed by further activation of STAT3 and extracellular signal-regulated kinase (ERK)1/2 through janus kinases (JAKs); however the importance of glycosylation of gp130 remains to be elucidated in cardiac myocytes. In this study, we examined the biological significance of gp130 glycosylation using tunicamycin (Tm), an inhibitor of enzyme involved in N-linked glycosylation. In cardiomyocytes, the treatment with Tm completely replaced the glycosylated form of gp130 with its unglycosylated one. Tm treatment inhibited leukemia inhibitory factor (LIF)-mediated activation of STAT3 and ERK1/2. Similarly, IL-11 failed to activate STAT3 and ERK1/2 in the presence of Tm. Interestingly, Tm inhibited the activation of JAKs 1 and 2, without influencing the expression of suppressor of cytokine signalings (SOCSs) and protein-tyrosine phosphatase 1B (PTP1B), which are endogenous inhibitors of JAKs. To exclude the possibility that Tm blocks LIF and IL-11 signals by inhibiting the glycosylation of their specific receptors, we investigated whether the stimulation with IL-6 plus soluble IL-6 receptor (sIL-6R) could transduce their signals in Tm-treated cardiomyocytes and found that this stimulation was unable to activate the downstream signals. Collectively, these findings indicate that glycosylation of gp130 is essential for signal transduction of IL-6 family cytokines in cardiomyocytes.

## Introduction

Accumulating evidence has demonstrated that interleukin-6 (IL-6) family cytokines play important roles in the maintenance of cardiac homeostasis [1]. Among IL-6 family cytokines, leukemia inhibitory factor (LIF), cardiotrophin-1 (CT-1), and IL-11, but not IL-6, have been reported to transduce their signals, such as signal transducers and activator of transcription (STAT)3 and extracellular signal-regulated kinase (ERK) 1/2 in cardiac myocytes [2], [3]. These cytokines bind their specific receptor α subunits that are expressed in cardiac myocytes and activate their common receptor subunit, glycoprotein 130 (gp130) [4]. Since the activation of gp130 is essential for the signal transduction of IL-6 family cytokines, the structure of gp130 molecule has been intensively investigated. As a result, structural biological studies have proposed the glycosylation sites on gp130 molecule [5]; however, the biological significance of the glycosylation of gp130 remains to be fully elucidated in cardiac myocytes.

Tunicamycin (Tm), an inhibitor of N-acetylglucosamine phosphotransferase, is widely used as an inducer of endoplasmic reticulum (ER) stress [6]. Tm treatment induces ER stress by inhibiting N-glycosylation of cell surface and secreted glycoproteins in the ER and Golgi. Interestingly, it was recently demonstrated that ER stress causes leptin resistance [7], [8]. Under the condition of ER stress, protein-tyrosine phosphatase 1B (PTP1B) plays important roles in the suppression of leptin signaling by inhibiting janus kinase (JAK) 2 activity and leptin fails to activate JAK2/STAT3 pathway in the presence of Tm. Though leptin receptor structurally resembles gp130, utilizing JAK/STAT pathway [9], the effects of Tm on gp130 signaling in cardiomyocytes remain to be fully addressed.

In this study, we examined the effects of Tm on the glycosylation of gp130 and its downstream signals in cardiac myocytes. Tm treatment completely replaced glycosylated gp130 with its unglycosylated form and inhibited STAT3 and ERK1/2 activation by IL-6 family cytokines in cultured cardiac myocytes. Based on these findings, we concluded that the N-glycosylation of gp130 is essential for the signal transduction through gp130.

## Materials and Methods

### Ethics statement

The experiments using animals were approved by the Institutional Animal Care and Use Committee of Graduate School of Pharmaceutical Sciences, Osaka University (Permit Number; DOUYAKU19-32-6). The care of all animals was performed in compliance with the Osaka University animal care guidelines.

### Cell culture

Neonatal rat cardiomyocytes were cultured as described previously [10]. In brief, after the rats were anesthetized with isoflurane, hearts were excised, minced and digested with solution containing 0.1% collagenase type IV and 0.1% trypsin to obtain a single-cell suspension. After preplating the cells on culture dish for 60 minutes, floating cells were used as cardiomyocytes. Isolated cardiomyocytes were cultured in Dulbecco’s Modified Eagles Medium (D-MEM); high glucose with L-Glutamine and sodium bicarbonate (Sigma-Aldrich) supplemented with 10% fetal bovine serum and bromodeoxyuridine (0.1 µg/mL).

To analyze the effects of Tm, cultured cardiomyocytes were treated with the indicated concentrations of Tm for the indicated hours. Cells were washed with serum free medium and used for experiments. Cells were stimulated with LIF (300 units/mL), IL-11 (20 ng/mL) or IL-6 (20 ng/mL) plus soluble IL-6 receptor (sIL-6R) (100 ng/mL) for 15 minutes.

### Immunoblot analyses

Immunoblot analyses were performed as described previously [11]. Cell lysates were prepared by adding SDS-PAGE sample solution to cells and boiled for 5 minutes. Proteins were separated by SDS-PAGE on polyacrylamide gels and transferred to polyvinylidene difluoride membranes (Millipore). The membrane was blocked with 2% skim milk for 1 hour, followed by incubation with the first antibody overnight at 4°C. Horseradish peroxidase (HRP)-conjugated antibody (Santa Cruz Biotechnology) was used as secondary antibody. The bands were detected by ECL system (GE Healthcare). Densitometric analyses of the detected bands were performed with Image-J software.

The first antibodies used for this study are as follows; anti-phospho-STAT3 (#9131), anti-phospho-ERK1/2 (#9101), anti-ERK1/2 (#9102), anti-phospho-JAK2 (#3771), anti-JAK2 (#3230) and anti-CHOP (#2895) antibodies were purchased from Cell Signaling Technology. Anti-STAT3 (sc-7179), anti-gp130 (sc-656), anti-phospho-JAK1 (sc-16773), anti-JAK1 (sc-7228), anti-LIF receptor (LIFR) (sc-659), anti-IL-11 receptor α (IL-11Rα) (sc-993) and anti-PTP1B (sc-1718) antibodies were obtained from Santa Cruz Biotechnology. Anti-GAPDH antibody (MAB374) was purchased from Millipore, and anti-Bip/GRP78 (610978) antibody was from BD Biosciences.

### Immunofluorescence microscopy

Cells were fixed with 3.7% formaldehyde in PBS and washed with PBS twice. Permeabilized with 0.2% TritonX-100 in PBS for 2 minutes, cells were stained with anti-gp130 antibody as a primary antibody for 1 hour at room temperature. After washed with PBS twice, Alexa Fluor488 anti-rabbit IgG (Molecular Probes) was applied for 1 hour at room temperature as a secondary antibody. Nuclei were also stained with Hoechst 33258 (Sigma). Cells were examined with confocal microscopes (Leica TCS SP5) and fluorescent microscopy (Olympus FSX100).

### PCR

Real time PCR with cDNA was performed to quantify mRNA as described previously [12]. Briefly, total RNA was prepared from neonatal rat cardiomyocytes with QIAzol reagent (QIAGEN) according to the manufacture’s protocol, and 1 µg of total RNA was subjected for first strand cDNA synthesis with Oligo (dT) primer (Invitrogen), dNTPs (Roche), RNase Inhibitor (TOYOBO) and Rever Tra Ace (TOYOBO). Using the synthesized cDNA, the expression levels of SOCS1, SOCS3, PTP1B and GAPDH were quantified by real time PCR using the SYBR Green Master Mix (Applied Biosystems) according to the manufacture’s protocol. The primers used in this study were shown in Table 1.

**Table 1:** The primers used in this study.

Genes	Direction	Sequence
CHOP	Forward	5′-GCACCTCCCAGAGCCCTCACTCTCC-3′
	reverse	5′-GTCTACTCCAAGCCTTCCCCCTGCG-3′
Grp78	forward	5′-GAAGGGGAGCAGAAAGCCCAT-3′
	reverse	5′-GCTTTGGTGAGGTTTGATCCGC-3′
SOCS1	forward	5′-CCGCTCCCACTCTGATTACC-3′
	reverse	5′-CGAAGCCATCTTCACGCTGA-3′
SOCS3	forward	5′-CGACGGAACCTTCCTTTGAGG-3′
	reverse	5′-TAGCCACGTTGGAGGAGAGAG-3′
PTP1B	forward	5′-TGCACAGCATGAGCAGTATG-3′
	reverse	5′-TGTGCCTTTTGTTCCTCCTC-3′
GAPDH	forward	5′-CATCACCATCTTCCAGGAGCG-3′
	reverse	5′-GAGGGGCCATCCACAGTCTTC-3′

### Statistical analysis

All data are presented as means ± S.D. Multiple comparisons were performed by One-way ANOVA with post-hoc multiple comparison test using SPSS software. *P*<0.05 was considered to be statistically significant.

## Results

### The treatment with tunicamycin completely inhibited the glycosylation of gp130 in cardiac myocytes

**Figure 1:**
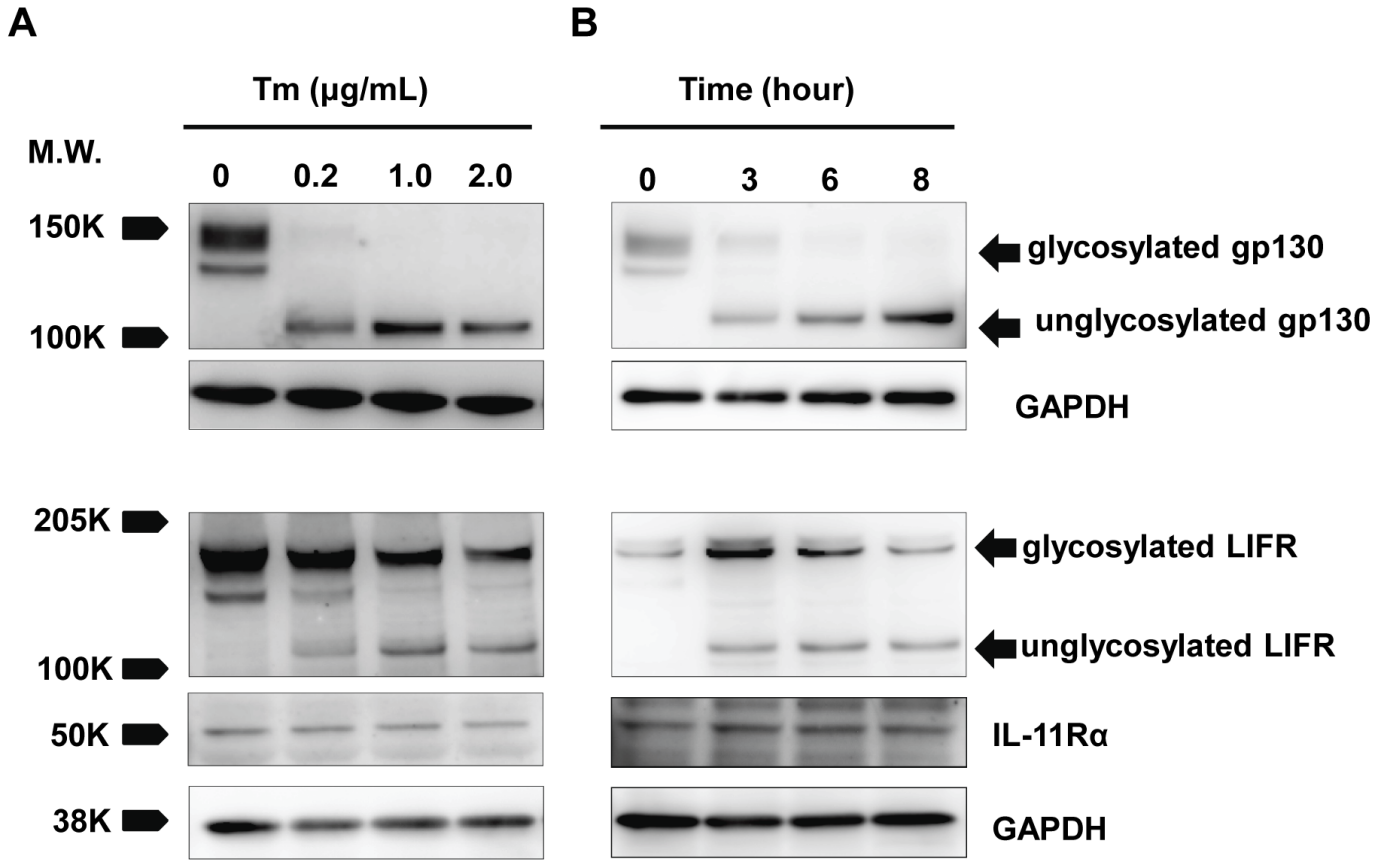
Tunicamycin inhibits the glycosylation of gp130 completely, but does those of LIFR and IL-11Rα partially. Neonatal rat cardiomyocytes were treated with the indicated concentrations of Tm for 8 hrs (A), or with 2 µg/mL of Tm for the indicated times (B), respectively. After each treatment, cell lysates were prepared and immunoblotted with anti-gp130, LIFR or IL-11Rα antibody, respectively. More than three independent experiments were performed with similar results and representative images were shown.

Since Tm is an inhibitor of N-acetylglucosamine phosphotransferase, we examined the effects of Tm on the glycosylation of gp130 in cultured cardiac myocytes. Cultured cardiomyocytes were incubated with various concentrations of Tm for 8 hours or with 2 µg/mL of Tm for the indicated times (0, 3, 6, 8 hours). The immunoblot analyses have demonstrated that Tm treatment reduced the molecular weight of gp130 in a dose- and time-dependent manner (Figure 1A and 1B). This reduced molecular weight (slightly bigger than 100 kDa) corresponds with that of its unglycosylated form, 101 kDa as previously reported [5]. These results indicate that Tm could completely replace glycosylated gp130 with its unglycosylated form, and then cells were pretreated with 2 µg/mL of Tm for 8 hours for further experiments. The inhibitory effect of Tm on the N-glycosylation of gp130 continued more than 16 hours after Tm washed out from the culture media in cardiomyocytes (Figure S1).

In addition, the effects of Tm on the glycosylation of LIFR and IL-11Rα were also proved because each of them heterodimerizes with gp130 respectively and activates downstream signals. As shown in Figure 1, Tm reduced the molecular weight of LIFR partially, whereas Tm did not affect the glycosylation of IL-11Rα. For LIFR, the molecular weight of reduced band is consistent with that in the previous report [13], indicating that Tm incompletely inhibited the glycosylation of LIFR in cardiac myocytes.

### Tunicamycin did not alter the localization of gp130

**Figure 2:**
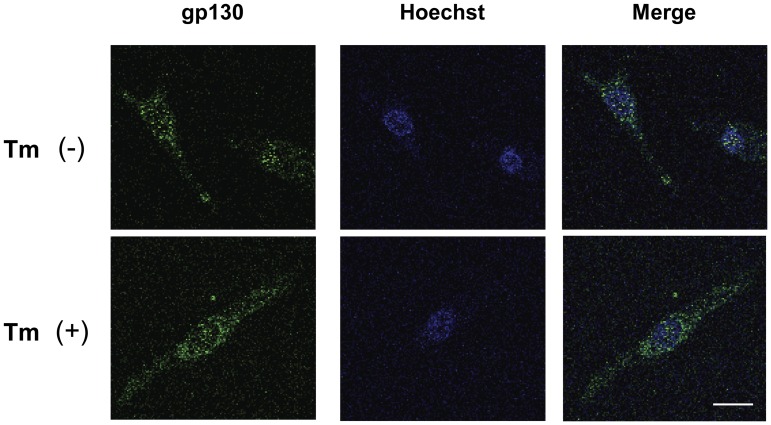
Tunicamycin does not influence the localization of gp130 in cardiomyocytes. Neonatal rat cardiomyocytes were treated with or without Tm (2 µg/mL) for 8 hours. Cultured cells were fixed and immunostained with anti-gp130 antibody or Hoechst for nuclei. Bar indicates 15 µm. Representative images were shown.

Previously, it was reported that the inhibition of gp130 glycosylation does not impair its translocation to cellular membrane [13]. Consistently, immunofluorescence microscopic analyses revealed that Tm does not alter the localization of gp130 (Figure 2).

### Tunicamycin inhibits STAT3 activation by IL-6 family cytokines in cultured cardiac myocytes

**Figure 3:**
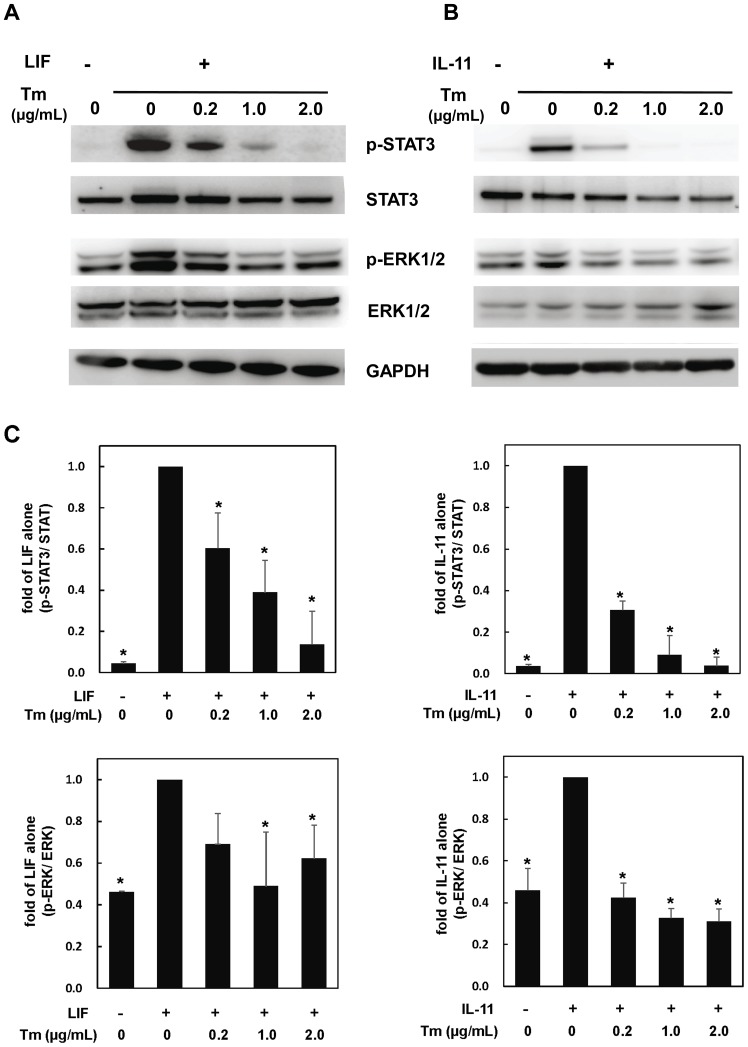
Tunicamycin inhibits the activation of STAT3 and ERK by LIF or IL-11, respectively. Cultured cardiomyocytes were pretreated with the indicated concentration of Tm for 8 hours, and stimulated with LIF (300 U/mL) (A) or IL-11 (20 ng/mL) (B) for 15 minutes. Activation of STAT3 and ERK1/2 was analyzed by immunoblotting with each phospho-specific antibody. Membranes were stripped and reprobed with anti-STAT3, anti-ERK1/2, or anti-GAPDH antibody, respectively. Representative images were shown (A and B). (C). For quantification, densitometric analyses for STAT3 or ERK1/2 phosphorylations were normalized with those of total STAT3 or total ERK, respectively. Values were converted based on that of each group treated with cytokine alone. Data were mean ± S.D. of three independent experiments. Dunnett test was performed for post-hoc multiple comparison test. *; *P*<0.05 versus cytokine alone.

**Figure 4:**
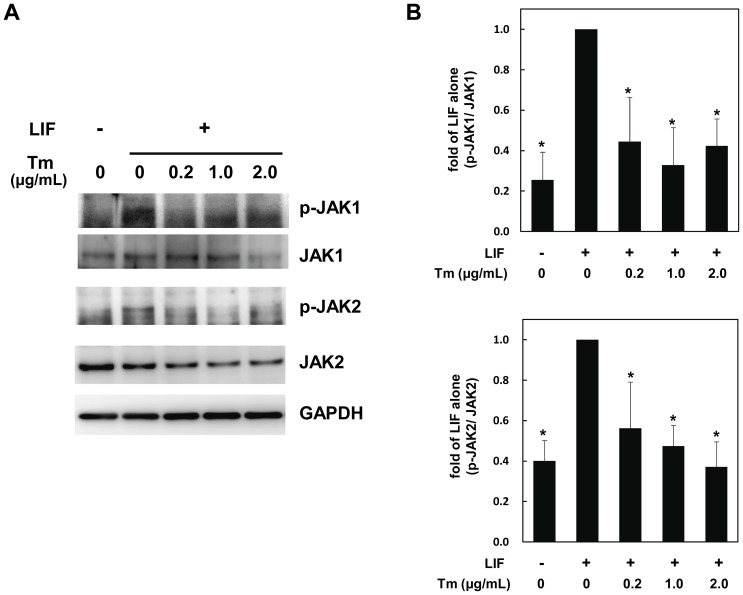
Tunicamycin suppresses the gp130-mediated activation of JAK1 and 2. Neonatal rat cardiac myocytes, pretreated with the indicated concentrations of Tm for 8 hours, were stimulated with LIF (300 U/ml) for 15 minutes. The activation of JAK1 and JAK2 was analyzed by immunoblotting with the phospho-JAK1 and phospho-JAK2 specific antibodies. Membranes were stripped and reprobed with anti-JAK1, anti-JAK2, or anti-GAPDH antibody, respectively. Representative images were shown (A). (B). For quantification, densitometric analyses for JAK1 and JAK2 phosphorylation were normalized with those of total JAK1 or total JAK2, respectively. Values were converted based on that of each group treated with LIF alone. Data were mean ± S.D. of three independent experiments. Dunnett test was performed for post-hoc multiple comparison test. *; *P*<0.05 versus LIF alone.

In order to examine the biological significance of gp130 glycosylation, cardiac myocytes were stimulated with LIF, an IL-6 family cytokine, in the presence of various concentrations of Tm. As reported previously [14], LIF activated STAT3 and ERK1/2; however, Tm inhibited LIF-mediated activation of these signaling pathways (Figure 3A). Recently, we reported that IL-11, which also belongs to IL-6 family cytokines, activates STAT3 and ERK1/2 through IL-11R that is expressed in cultured cardiac myocytes [3]. Therefore, we examined the effects of Tm on IL-11 activation of STAT3 and ERK1/2. As is the case with LIF, IL-11-induced activation of STAT3 and ERK1/2 was inhibited by Tm in a dose-dependent manner (Figure 3B). As shown in Figure 3C, quantitative analyses exhibit Tm significantly inhibited the activation of STAT and ERK1/2 by these two IL-6 family cytokines, respectively. Next, we checked whether Tm influences JAKs activities using phospho-JAK1 and phospho-JAK2 specific antibodies. LIF enhanced the phosphorylation of both JAK1 and JAK2, which was suppressed by Tm significantly (Figure 4). These data indicate that Tm inhibits JAKs/STAT3 activation by IL-6 family cytokines in cardiac myocytes.

### Tunicamycin inhibited JAK/STAT3 pathway downstream of gp130 independently of PTP1B and SOCSs

**Figure 5:**
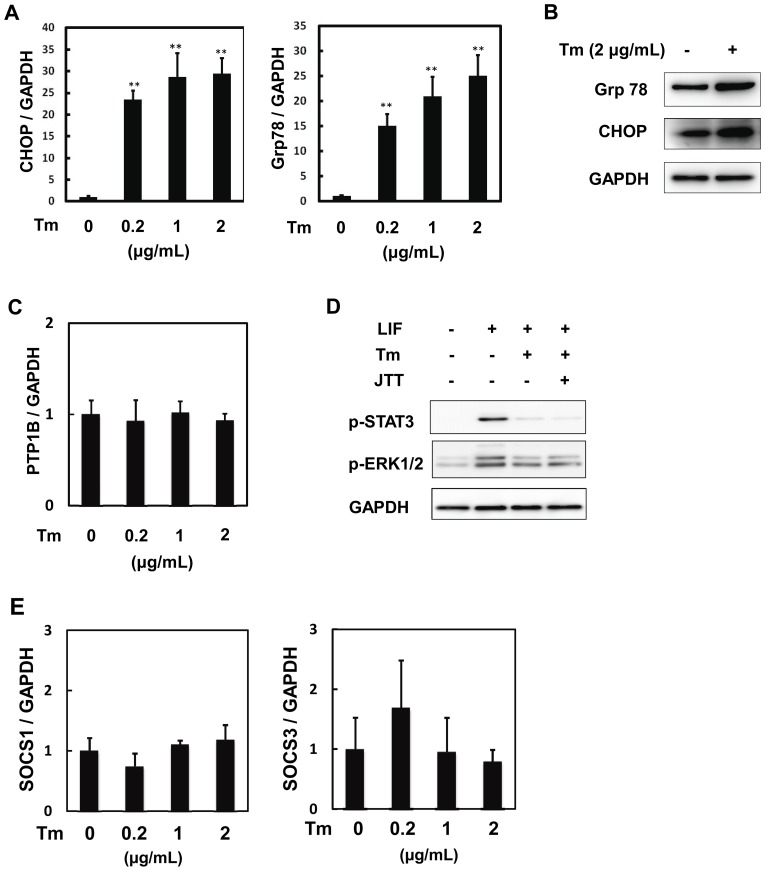
Tunicamycin inhibits JAK/STAT3 pathway downstream of gp130 independently of ER stress, PTP1B and SOCSs. Neonatal rat cardiac myocytes were cultured with the indicated concentrations of Tm for 8 hours. Total RNA was prepared and applied for reverse transcription. Real time PCR system was used to detect the mRNA expression of CHOP (A), Grp78 (A), PTP1B (C), SOCS1 and 3 (E) as described under ‘Material and Methods’. The expression level of each gene was normalized with that of GAPDH, an internal control, and represented as value of fold induction relative to those of each non-treated group with Tm (control). Data were shown as mean ±S.D. (n = 3). **; *P*<0.05 versus control at the multiple comparison test. After cardiac myocytes were pretreated with or without Tm (2 µg/mL) for 8 hours, cells were washed with medium and incubated for more 15 hours. Afterward, cells lysates were prepared for immunoblotting analyses with anti-CHOP, anti-Grp78 and GAPDH antibody. Experiments were repeated three times with similar results and representative data are shown in (B). Cardiac myocytes were pretreated with or without Tm (2 µg/mL) for 8 hours in the presence or absence of JTT551 (JTT), PTP1B inhibitor, and stimulated with LIF (300 U/I) for 15 minutes. Activations of STAT3 and ERK1/2 were analyzed by immunoblotting with anti-phospho-STAT3, anti-phopho-ERK1/2 and anti-GAPDH antibody. Representative images were shown in (D).

Previously, ER stress was reported to inhibit JAK/STAT3 pathway in leptin signaling pathway through PTP1B [7], [8] In cardiac myocytes, Tm increased the expression of the marker genes for ER stress, such as C/EBP-homologous protein (CHOP) and glucose-regulated protein 78 (Grp78) (Figure 5A & 5B). Therefore, we examined whether Tm induced PTP1B and found that the expression of PTP1B mRNA was not influenced by Tm (Figure 5C). Consistently, Tm inhibited LIF-mediated activation even in the presence of JTT551, a PTP1B inhibitor [15] (Figure 5D). We also examined the expression of SOCS1 and 3, but the expression of these genes was not influenced by Tm (Figure 5E). These data indicate that Tm inhibits the gp130-mediated activation of JAK/STAT3 differently from leptin-induced STAT3 activation.

### Treatment with IL-6 plus sIL-6R failed to stimulate STAT3 and ERK1/2 in the presence of Tunicamycin

**Figure 6:**
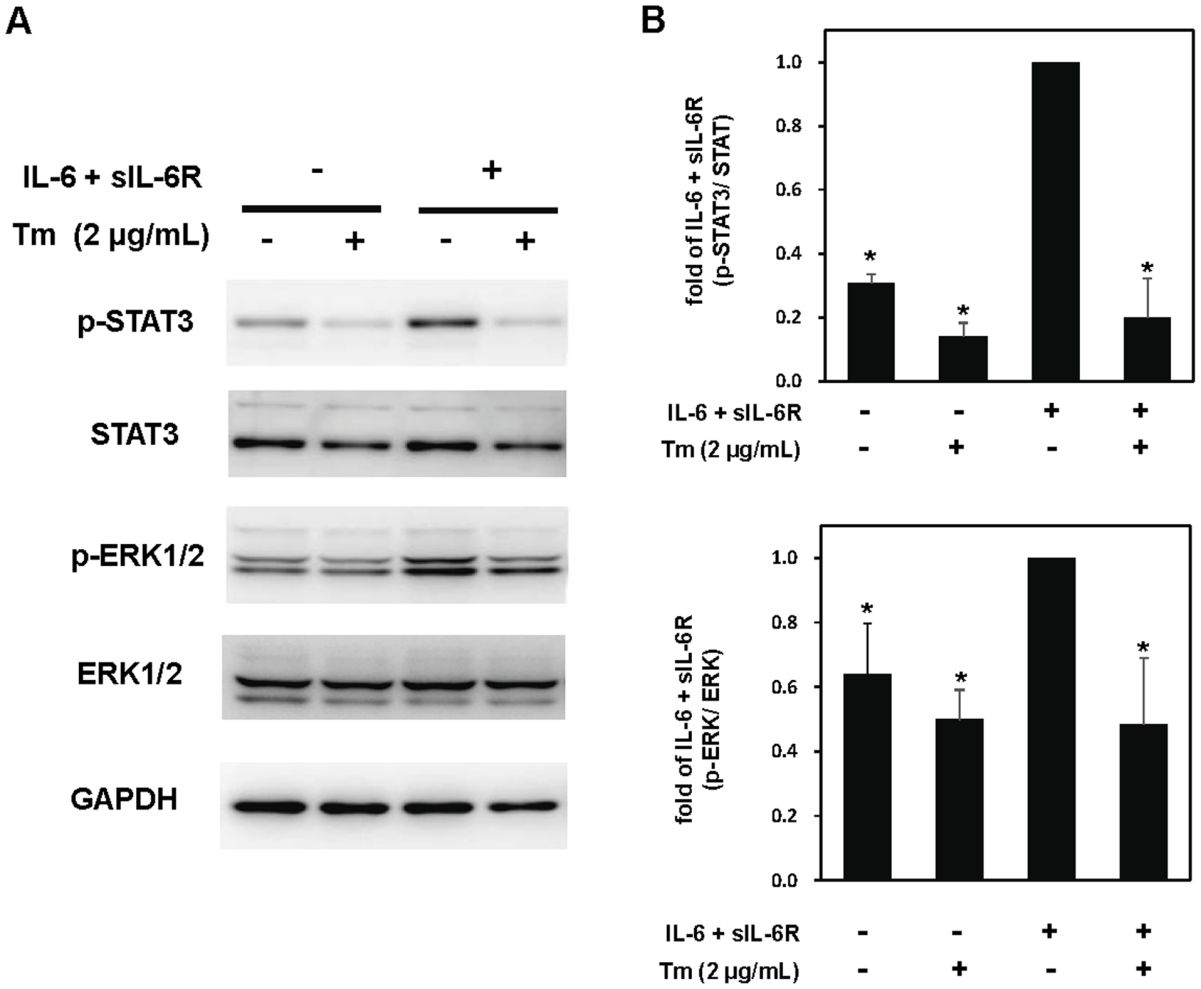
The combined treatment with IL-6 and sIL-6R fails to activate STAT3 in the presence of tunicamycin. Neonatal rat cardiac myocytes, pretreated with or without Tm (2 µg/mL) for 8 hours, were stimulated with IL-6 (20 ng/mL) plus sIL-6R (100 ng/mL) for 15 minutes. Activation of STAT3 and ERK1/2 were analyzed by immunoblotting with each phospho-specific antibody. Membranes were stripped and reprobed with anti-STAT3, anti-ERK1/2, or anti-GAPDH antibody, respectively. Representative images were shown (A). (B). For quantification, densitometric analyses for STAT3 or ERK1/2 phosphorylation were normalized with those of total STAT3 or total ERK, respectively. Values were converted based on that of each group treated with cytokine alone. Data were mean ± S.D. of three independent experiments. Dunnett test was performed for post-hoc multiple comparison test. *; *P*<0.05 versus the combination of IL-6 and sIL-6R alone.

We have demonstrated that Tm inhibited activation of STAT3 and ERK1/2 by LIF and IL-11; however, we cannot completely rule out the possibility that the glycosylation of the endogenous LIFR and IL-11R might be modulated by Tm and lose the capacity of activating gp130, though the molecular weight of some fraction of LIFR and/or IL-11R was not apparently affected by Tm (Figure 1). Therefore, to exclude the effects of Tm on glycosylation of the endogenous receptor α subunits, we treated cultured cardiomyocytes with IL-6 and sIL-6R with or without Tm pretreatment. Since sIL-6R is an agonistic receptor, the combined treatment with IL-6 and sIL-6R activated STAT3 and ERK1/2 in the absence of Tm; however, this combined treatment failed to transduce STAT3 and ERK1/2 signalings when the cardiomyocytes were pretreated with Tm (Figure 6).

## Discussion

In this study, we examined the effects of Tm on gp130 signaling in cultured cardiomyocytes. The treatment with Tm inhibited the activation of JAK/STAT3 in response to LIF or IL-11 in cardiomyocytes. Tm induced ER stress in cardiomyocytes; however, PTP1B is not involved in the inhibition of JAK/STAT3 by Tm, suggesting that gp130 signaling was suppressed by Tm differently from leptin signal.

The treatment with Tm replaced the N-glycosylated gp130 with its unglycosylated form completely, whereas Tm partially inhibited the glycosylation of LIFR and Tm did not affect that of IL-11Rα. In addition, the inhibitory effect of Tm on unglycosylated gp130 continued more than 15 minutes even after Tm was washed out from the medium. Under these conditions, the unglycosylated form of gp130 failed to activate STAT3 and ERK1/2 in response to the stimulation with LIF, IL-11 or IL-6 plus sIL-6R. Based on these findings, it is concluded that N-glycosylation of gp130 is essential for the signal transduction of gp130 system.

Previously, Yanagisawa et al. evaluated the functional role of N-glycans of gp130 in mouse embryonic neural stem cells (NSCs) using Tm (13). In NSCs treated with Tm, some fraction of gp130 was detected as its unglycosylated form. Unglycosylated gp130 was translocated to the cell surface but did not form a heterodimer with LIFR, analyzed by immune-precipitation assays. In spite of its loss of heterodimerization ability with LIFR, LIF stimulation activated STAT3 and ERK in the presence of Tm to the same level with their activation in the absence of Tm. This discrepancy might be explained by the limitation of their experimental system; significant fraction of gp130 was still expressed as its N-glycosylated form in NSCs even in the presence of Tm. Another possibility is that LIFR was also unglycosylated by Tm and might be functionally modified, resulting in transducing LIF signal independently of gp130. In this study, when we successfully replaced N-glycosylated gp130 with the unglycosylated form completely, LIFR was not completely replaced with the unglycosylated forms and IL-11Rα was not affected. Under this glycosylation state of each receptor, the signals of JAK/STST3 and ERK1/2 by LIF or IL-11 were inhibited. Besides these results, we demonstrated that the glycosylation is essential for gp130 signaling by using IL-6 and sIL-6R, which form ligand/receptor complex and then proceed trans-signaling.

In contrast to our results, Waetzig et al reported that N-glycosylation is not essential for the signal function of gp130 by using the gp130 mutant with amino acid substitution from Asn to Gln at N-glycosylation sites (16). The loss of N-glycosylation in gp130 molecule reduces its stability but retains the ability to activate STAT3 in response to the agonistic complex of IL-6 and sIL-6R. Since amino acid substitutions could result in the intramolecular conformational changes in mutant gp130, it might be difficult to address the necessity of N-glycosylation by amino acid substitution method. Moreover, the authors used the agonistic complex of IL-6 and sIL-6R as hyperactive IL-6 (17). These approaches might artificially potentiate the mutant gp130 signaling function, though further studies would be required.

Previously, it was demonstrated that Tm inhibits leptin-mediated JAK2/STAT3 pathway through ER stress (7, 8). Under the ER stress, activation of JAK2 is inhibited by PTP1B. In cardiac myocytes, we have also confirmed that Tm induces ER stress; however, PTP1B is unlikely to be involved in Tm-mediated inhibition of STAT3 activation by IL-6 family. Indeed, PTP1B inhibitor did not recover STAT3 activity in the presence of Tm. Moreover, PTP1B specifically inhibits JAK2 activity, while IL-6 family cytokines activate STAT3 though both JAK1 and JAK2 (14).

Recently, the activation of the hexosamine biosynthesis pathway (HBP), which converts glucose to UDP-N-acetylglucosamine (GlcNAc) for N- and O-glycosylation of proteins, has been reported to protect various biological insults, such as ER stress, ischemia/reperfusion injury in heart and Tm toxicity (18, 19). Interestingly, supplementation with GlcNAc to worms led to Tm resistance (18). It is not clear whether GlcNAc or its metabolites could affect directly N-glycosylation, which maintains effective protein folding and ER proteostasis, to regulate cellular protein homeostasis. Further research on the crosstalk between HBP and gp130/JAKs/STAT signalings might provide insights into the molecular mechanisms of the onset of heart failure.

In conclusion, pharmacological approach using Tm, an inhibitor of enzymes involved in N-glycosylation, has revealed that N-glycosylation of gp130 is essential for its signal functions. Since gp130 signaling pathway plays crucial roles in the maintenance of cardiac homeostasis, the disturbance of its N-glycosylation might cause cardiovascular diseases.

## Supporting Information

Figure S1 **The reversibility of unglycosylated gp130 by Tm in cardiomyocytes.** Treated with or without Tm (2 µg/mL) for 8 hours, neonatal rat cardiac myocytes were washed twice with serum free medium and incubated again for the indicated times. Cells lysates were applied for immunoblotting analysis with anti-gp130 antibody to detect the reversibility of N-glycosylation of gp130. Representative images are shown.
(TIF)
