# Correction: The Inhibition of N-Glycosylation of Glycoprotein 130 Molecule Abolishes STAT3 Activation by IL-6 Family Cytokines in Cultured Cardiac Myocytes

**DOI:** 10.1371/journal.pone.0122930

**Published:** 2015-03-31

**Authors:** The PLOS ONE Staff

The author’s initial, TM, is missing from the funding statement. The correct funding statement is given below.

This study was supported by a grant-in-aid for scientific research from the Japan society for the promotion of science; YF, HN, TM, MM. This study was supported by MEXT/JSPS KAKENHI Grant Numbers 23390057, 26293054, 25860061 and 22590160. The funders had no role in study design, data collection and analysis, decision to publish, or preparation of the manuscript.
