# Supplementary figures and images for: The Inhibition of N-Glycosylation of Glycoprotein 130 Molecule Abolishes STAT3 Activation by IL-6 Family Cytokines in Cultured Cardiac Myocytes
Source: PLoS One. 2014 Oct 23;9(10):e111097. doi: 10.1371/journal.pone.0111097 (PMC4207791; doi:10.1371/journal.pone.0111097)

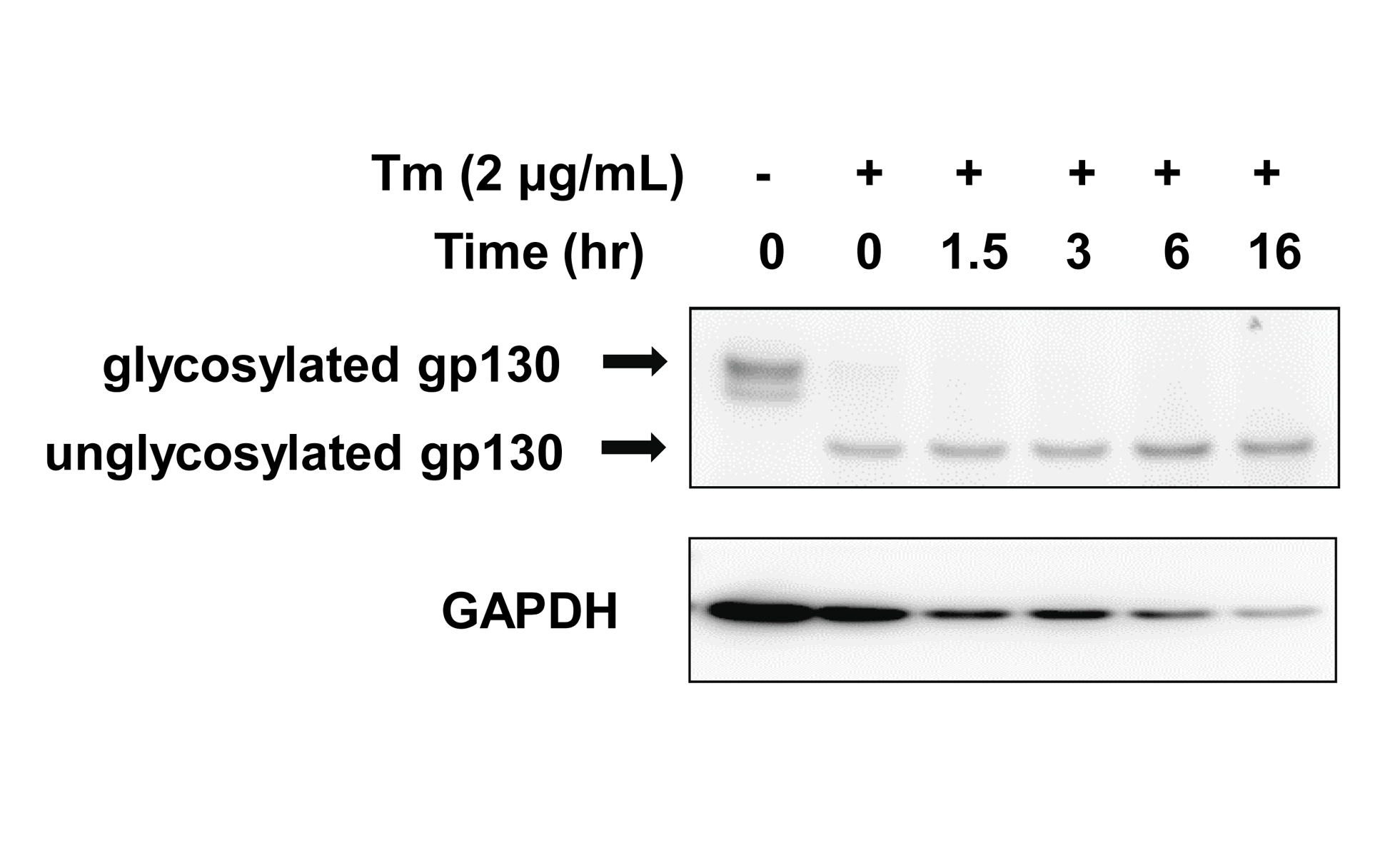

Supplement: Figure S1 — The reversibility of unglycosylated gp130 by Tm in cardiomyocytes. Treated with or without Tm (2 µg/mL) for 8 hours, neonatal rat cardiac myocytes were washed twice with serum free medium and incubated again for the indicated times. Cells lysates were applied for immunoblotting analysis with anti-gp130 antibody to detect the reversibility of N-glycosylation of gp130. Representative images are shown. (TIF) [file pone.0111097.s001.tif]
